# Outcomes of a Telephonic Postnatal Intervention for Mothers and Babies in Mopani District, Limpopo, South Africa

**DOI:** 10.3389/fgwh.2022.876263

**Published:** 2022-05-09

**Authors:** Chipo Mutyambizi, Jackie Dunlop, Rendani Ndou, Helen Struthers, James McIntyre, Kate Rees

**Affiliations:** ^1^Anova Health Institute, Johannesburg, South Africa; ^2^Division of Community Paediatrics, School of Paediatrics, Faculty of Health Sciences, University of the Witwatersrand, Johannesburg, South Africa; ^3^Division of Infectious Diseases and HIV Medicine, Department of Medicine, Faculty of Health Sciences, University of Cape Town, Cape Town, South Africa; ^4^School of Public Health and Family Medicine, Faculty of Health Sciences, University of Cape Town, Cape Town, South Africa; ^5^Department of Community Health, School of Public Health, University of the Witwatersrand, Johannesburg, South Africa

**Keywords:** postnatal care, mother and child health, South Africa, healthcare access, randomized controlled trial (RCT)

## Abstract

**Background:**

The postnatal period is a critical period for the health of both mother and infant. Studies show that postnatal care reduces neonatal mortality and other adverse mother and child health outcomes. While the World Health Organization recommends four postnatal care contacts, South African guidelines only specify three, excluding a 7-14-day post-birth contact. This study aimed to assess whether a telephonic contact at 7-14 days following delivery had any effect on use of additional postnatal services.

**Methods:**

A randomized controlled trial design was used to address the study objectives. Two groups of new mothers were randomly allocated to either receive the 7-14-day telephonic contact or not from a research nurse. Data for this study was collected at Maphutha L Malatjie Hospital (MLMH). Descriptive analysis was performed first, then a multivariable logistic regression analysis was conducted to assess the factors associated with access to other health care services.

**Results:**

A total of 882 mothers were recruited, 854 (97%) were classified as high risk, 28 (3%) were classified as low risk. 417 (49%) of the high risk received the 7-14-day call (intervention group) whilst the remainder of 437 (51%) from the high risk plus all mothers classified as low risk (28) did not receive the call (control group). 686 (78%) of all mothers received the 3 month follow up call. The call showed that 17 mothers from the control group and 10 mothers from the intervention group accessed other healthcare services. We find that hypertension (3.28; 1.06 −10.10), mental health risk (2.82; 1.25 −6.38), PV bleeding during pregnancy (18.33; 1.79–187.61), problem during labor (4.40; 1.280–15.13) were positively associated with access to other health services, with statistically significant associations (*p*-value < 0.05). We found statistically insignificant associations between receiving the 7-14-day call and accessing other health care services.

**Conclusion:**

The 7-14-day call had no statistically significant impact on access to other health services, however, high levels of satisfaction with the call may point to an unmet need for care at this time. It is important to investigate other innovative solutions to postnatal care improvement in South Africa.

## Background

In South Africa, maternal mortality rate (MMR) was estimated to be 119 per 100 000 live births in 2017, and neonatal morality rate (NMR) was estimated at 12 per 1,000 live births in 2019/2020 (1). According to the Sustainable Development Goals, the MMR should be <70 per 100 000 live births (SDG indicator 3.1), and NMR below 12 per 1,000 live births (SDG indicator 3.2) (2). The postnatal period is a very vulnerable time for a mother and her new infant and is defined by the World Health Organization (WHO) as the period from delivery until 6 weeks later (3). As a notable proportion of maternal and neonatal deaths occur within this period (3), health care is critical to the survival of both the mother and infant (3–5). Globally 2.4 million infants died within their first month of life in 2019 (17 deaths per 1,000 live births), accounting for 47% of all under-5 deaths (6). Close to three quarters of these neonatal deaths occur within the first week of life (6). Timely postnatal health contacts offer the mother an opportunity to receive health checks and information on how to take care of her baby (7). The South African Demographic and Health Survey 2016 reports that the percentage of women between the ages of 15 to 49 years who received postnatal care within 2 days of giving birth, was 83.6% in 2016, which had improved from 74.3% in 2014 (7). Unfortunately, on-going social inequality in South Africa drives the neonatal mortality rate within the country. Despite the recorded improvements in neonatal health outcomes, studies have shown race, age, income and urban vs. rural area disparities in access to and utilization of maternal and postnatal health care. (7, 8). Evidence from South Africa shows that the proportion of babies with a postnatal check during the first 2 days of birth was highest amongst those within the highest household wealth quantile and lowest amongst those within the lowest household wealth quantile (5, 7).

Previous studies have assessed the effects of postnatal care on newborn outcomes (9–11). A study conducted in Zambia found that a midwife home visiting programme at day 3, 7, 28 and 42 days after birth helped the mother to identify health problems and utilize health services when compared to mothers who received fewer postnatal visits (10). A Ugandan-based study showed the potential of community health workers (CHWs) in influencing essential newborn care practices and health care-seeking (11). It is estimated that women who receive midwife-led continuity of care, during pregnancy, birth and the post-partum period are 16% less likely for their baby to die (6). Promoting postnatal care is important for the advancement of other goals such as poverty reduction and equity and is a good measure of health system functionality.

Given how critical the postnatal phase is, the WHO provides guidelines on timelines of postnatal contacts and the content of these postnatal contacts (3). The WHO recommends four postnatal care contacts are delivered to newborns and their mothers through home or health facility visits (3). These visits are recommended to take place on the day of delivery, between day 3 to 6, between 7-14-days and at 6 weeks after birth (3). Due to limited resources, South African guidelines do not specify a 7-14-day postnatal clinic visit (12). Identifying women who are at increased risk of postnatal complications may help in guiding closer postnatal follow-ups for mothers (13). Using data from a randomized controlled trial, this study sought (1) to determine the effect of an additional postnatal contact during the 7-to-14-day period, on mother's utilization of additional healthcare services (2) determine what other factors are associated with additional service utilization and (3) provide a description of the post discharge deaths.

## Methods

The study methods and results section adhere to the Consolidated Standards of Reporting Trials (CONSORT) guidelines. The guidelines provide a minimum set of reporting items aimed at improving the quality of randomized controlled trial (RCT) reporting (14).

### Study Setting

**Figure 1:**
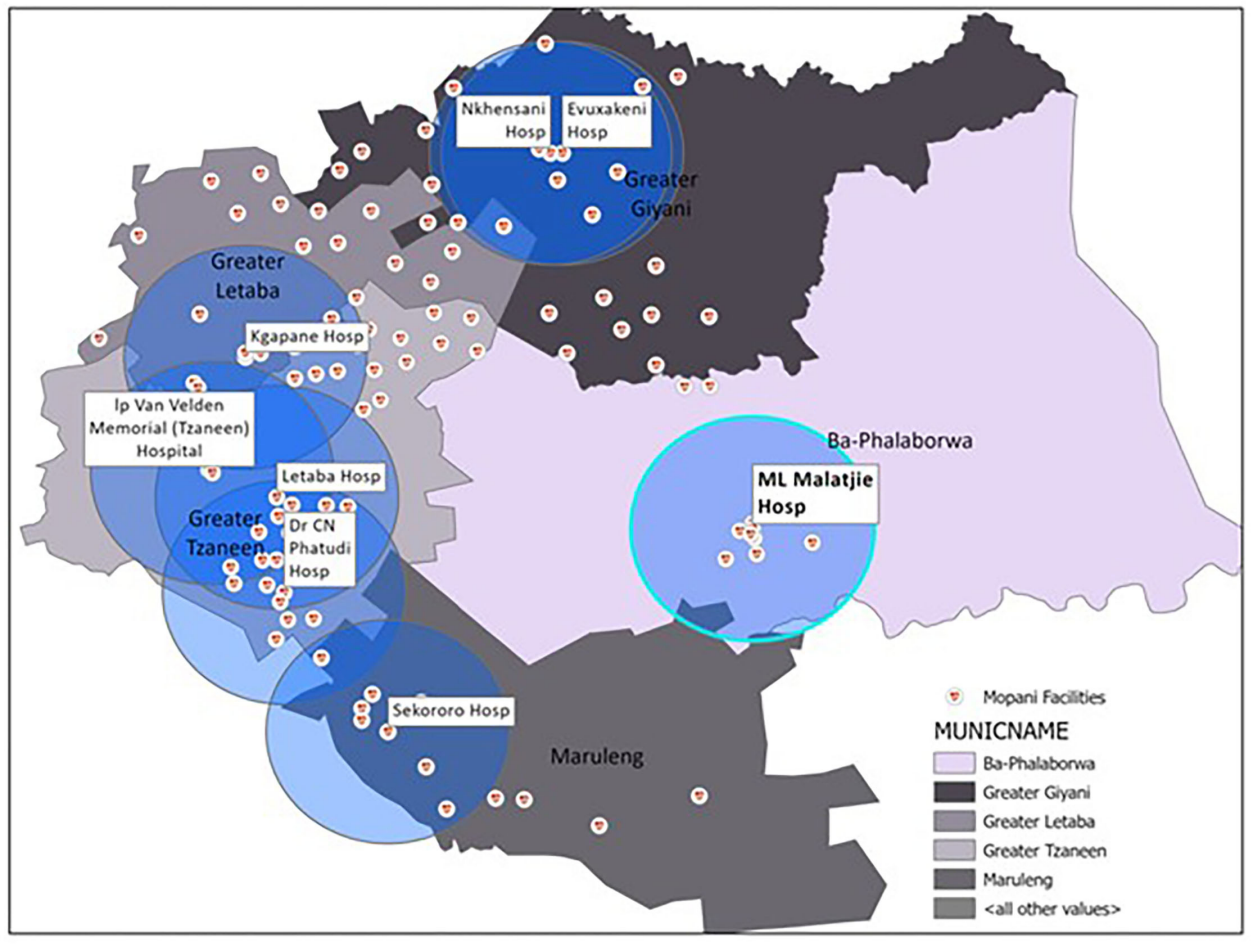
Health facilities in Mopani district.

This study was conducted in Mopani District, which is one of five districts within Limpopo province, in South Africa. Among the five district municipalities, Mopani has the third largest population at approximately 1.2 million (15). The district is sparsely populated, with most of its population residing in the rural areas, consisting of 55% female and 49% between the ages of 0 to 19 years old (16). The district is made up of five local municipalities (Ba-Phalaborwa, Greater Giyani, Greater Letaba, Greater Tzaneen and Maruleng) (see Figure 1) (17) and its major economic activity is mining (30%) followed by community services (22.6%) and trade (14.6%) (16). Statistics on the social determinants of health within the district indicate that the district has 49.2% female headed households, 39.4% unemployment rate and 90.7% formal dwellings (18). Healthcare within the district is provided by a mix of private and public facilities and it is estimated that private medical scheme coverage for the district is 6.7% (18), lower than the national average of 17.2% (19). Public health care is provided via a hierarchical referral system consisting of community-based systems at the bottom of the pyramid and central hospitals at the top.

Data for this study was collected at Maphutha L Malatjie Hospital (MLMH). It is a district level hospital, and is the only hospital located in the sub-district of Ba-Phalaborwa (see Figure 1). As per the Guidelines for Maternity Care in South Africa, pregnancies at higher risk of complication are referred to district hospitals for antenatal care and delivery. However, this referral system often does not function as planned and many mothers opt to deliver in the hospital despite not being high risk, as there is a perception of improved care and services available (20). Approximately 10% of the district's deliveries and 79% of the sub-district's deliveries occur at this hospital. The hospital was selected for the study because it is the only hospital in Ba-Phalaborwa and the majority of the sub-district's deliveries occur there.

The study formed part of the Mphatlalatsane Project, which is a collaboration of the South African Medical Research Council, National Department of Health, and the Clinton Health Access Initiative (CHAI) and aims to improve the quality of sexual and reproductive health in the Mopani District, Limpopo.

### Study Design and Instruments

A randomized controlled trial design was used to address the study objectives. The study made use of the following tools: a risk assessment tool, 7-to-14-day check tool, a follow up tool, 3 months follow up tool and a file review.

#### Risk Assessment (RA)

The research nurse made use of this tool to conduct risk assessments of MIPs. Face to face interviews were conducted at the time of discharge from MLMH, after delivery. The research nurse made use of a private space close to the maternity ward to conduct the face-to-face interviews with the mothers. As there is no known validated tool for identifying postnatal risk of complication following discharge the RA tool was developed by combining known risk factors of antenatal, intrapartum and postnatal complications. A risk assessment tool for perinatal depression and anxiety developed by the Perinatal Mental Health Project was also included. Those with a single identified risk factor were classified as high risk and randomly allocated into two groups (1) those that received the additional postnatal contact at between 7 to 14 days (intervention) and (2) those that did not receive the call (non-intervention). Those that were classified as low risk did not receive the 7-to-14-day call and were included in the non-intervention group during the analysis. Those who did not receive the call received the South African standard of postnatal care which includes a 3-6 day and 6-week postnatal facility visit and were able to seek additional care through standard pathways.

#### 7-to-14-Day Call

The research nurse contacted mothers telephonically between days 7 to 14 after delivery. This took place in addition to the South African standard of postnatal care, including the 3–6-day postnatal visit. During the call the research nurse administered a checklist that included a review of the care that the MIPS received at their 3–6 days facility visits, an assessment of maternal postnatal complications and neonatal danger signs, and a validated tool to screen for postpartum depression in pregnancy. Any MIP with problems or complications identified during this call was referred to the appropriate level of care by the research nurse.

#### Follow Up

A week after the 7-to-14-day call, the research nurse would follow up on all MIPs with postnatal problems or complications identified at the 7-to-14-day call. The objective of this call was to ensure that the MIP received services for the issues identified during the 7-to-14-day call and that the issues were resolved.

#### Three Month Follow Up

A telephonic follow up was conducted by the research nurse with all study participants (high risk and low risk) enrolled at the beginning of the study. The purpose of the call was to determine whether MIPs experienced any complications, had any additional interactions with health services (beyond the 3-6 day and 6 weeks' postnatal visits) (further referred to as access to other health services) and their self-reported resolution of postnatal problems.

#### File Review

A file review was then conducted at MLMH for all MIPs who accessed further care outside of the standard South African postnatal care. This was done to gather information on the complaint, diagnosis and treatment prescribed. In cases were MIPs accessed additional care from private hospitals such records were not available in the government sector hospital files and could therefore not be included in the analysis.

### Ethical Approval

Ethical approval for data collection was obtained from the Human Sciences Research Council Research Ethics Committee REC 5/21/08/19 and the Limpopo Provincial Health Research Committee (ref: LP-201909-012). Each mother also provided written informed consent to participate in the study.

### Sampling and Data Collection

There are an estimated 1000 deliveries in a 6-month period at MLMH. We determined the appropriate sample size for detecting a difference between the proportion of mothers who accessed other healthcare services in the intervention vs. the control group. The sample size was estimated at 683, assuming a sample proportion in the intervention group of 15% and 10% in the control group, a confidence interval of 95% and power of 80%. We added another 10% to this number to account for the possibility of refusals to participate. Postnatal women were excluded from the study if (1) they refused to participate (2) lived outside the Ba-Phalaborwa sub-district (3) were <18 years old and not accompanied by their parent or legal guardian.

Data collection was conducted from 3 August 2020 to 29 January 2021 during working hours (Monday to Friday). All mothers discharged from the postnatal ward of MLMH during the study period were invited to participate. No incentives or inducement was offered to the mothers to participate in the study. Face to face interviews and telephonic interviews were conducted with consenting mothers by an experienced research nurse based at the hospital during the study period. Interviews were conducted in the respondents preferred language to overcome language barriers and ensure participants were comfortable and confident with their response. Quality checks for all data collected by the research nurse was done once a week during the study.

To keep similar numbers of mothers in the intervention and control groups, block randomization was applied. High risk MIP's study identifiers (IDs) were aggregated in blocks of ten, according to when they were recruited, and then randomized into two: (1) MIPs receiving the additional postnatal contact at 7 to 14 days intervention (2) MIPs receiving the standard South African postnatal care with no additional postnatal contact at 7 to 14 days. Mothers were then informed whether to expect a 7-14-day call prior to being discharged from the hospital.

### Measures

The main outcome variable in this study is access to other healthcare services. At three months mothers were asked if the MIP accessed any other health services during the three months post-delivery. This was coded into a binary variable. Secondary measures used to describe the postnatal period after hospital discharge were post-discharge infant deaths following discharge and maternal experiences during the prescribed 3-6-day visit.

The 7-to-14-day call was the main independent variable for this study. Those who received the call were part of the intervention group and those who did not receive it were within the non-intervention group. Covariate variables included in our analysis were taken from the risk assessment and 3 months follow up tool. Demographic variables, health related variables, pregnancy factors, delivery and baby factors considered for the analyses were as follows: For the demographic variables we included age, employment, and education. Age was measured in years and categorized as 18 years and below, 19 to 24 years, 25 to 34 years, and 35 years and older. Employment was included as a binary variable. Education was categorized as follows: –did not complete or completed grade 12

Health related factors included HIV status, hypertension during pregnancy (gestational hypertension or chronic hypertension before pregnancy), maternal pre-existing chronic conditions, and mental health risk factors. These were included as binary variables. Pregnancy related factors included having four or more pregnancies, PV bleeding during pregnancy and no antenatal care (not booked). These were also included as binary variables in the analysis. Delivery related factors included delivery out-of-facility, problems during labor, preterm labor, cesarean delivery, perineal problems. Mothers were asked if they experienced any complications, and these were also checked in the discharge summary. All delivery related factors were included as binary variables. All baby factors, which included low birthweight (<2500 g), high birthweight (4000 g or over) and resuscitation at birth were included as binary variables.

### Data Analysis

The analysis was restricted to mothers who received the 3 months follow up call. Statistical analysis was conducted in STATA 14. Descriptive analysis and multivariable logistic regression analysis were conducted to assess the factors associated with access to other health care services amongst the MIPs. Variables were evaluated as potential determinants of access to health care through bivariate logistic regression analysis. Collinear variables were omitted from the analysis and explanatory variables that had an association *p*-value of ≤ 0.3 were included for the final multivariable logistic regression model.

## Results

**Figure 2:**
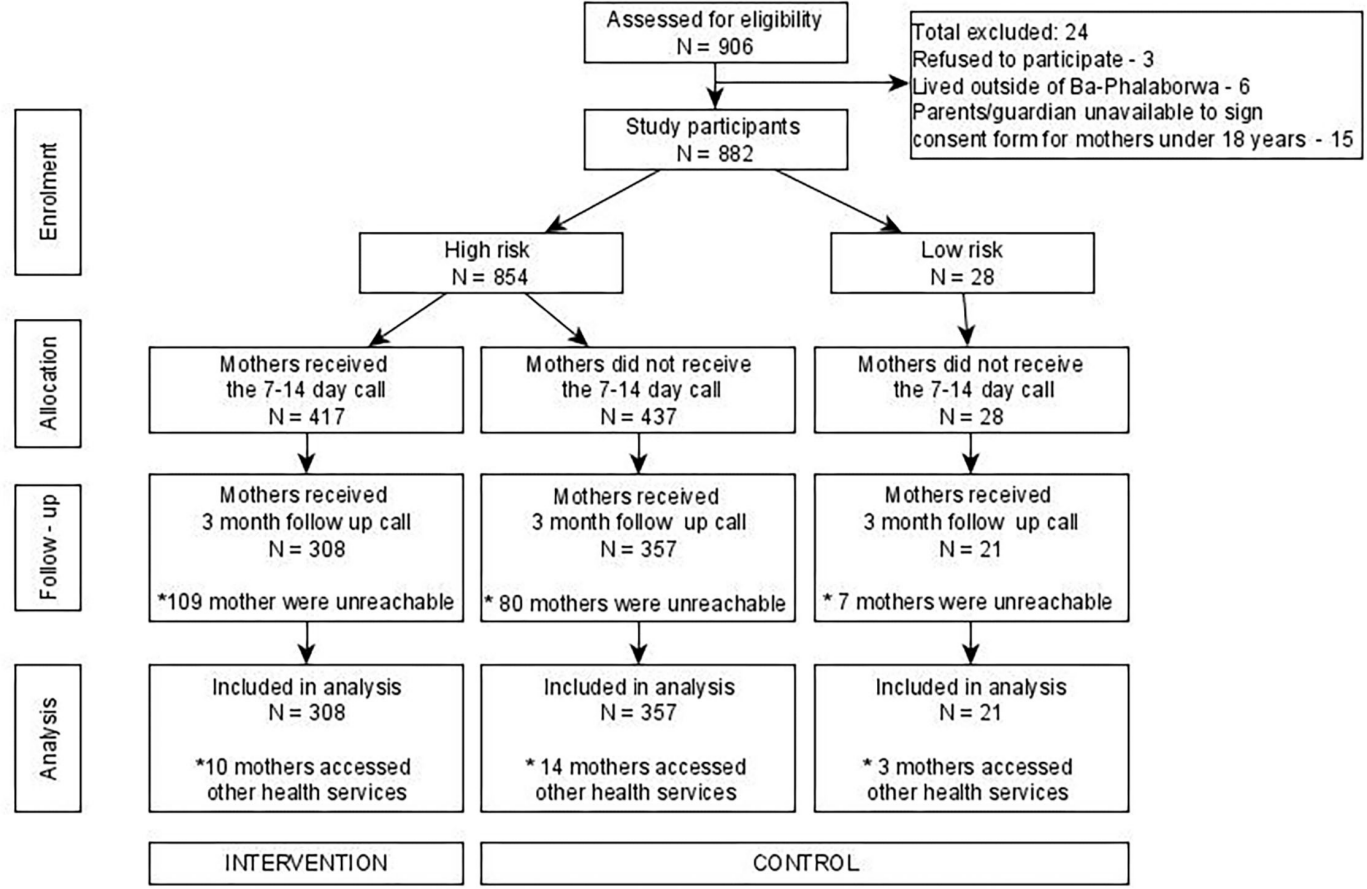
Study flow chart.

A total of 906 mothers were invited to take part in the study of which 24 mothers did not meet the inclusion criteria (see Figure 2). A total of 882 mothers completed the risk assessment tool (see Figure 2). Of these, 854 (97%) were classified as high risk. Amongst those classified as high risk 417 (49%) received the 7-to-14-day call from the research nurse (intervention group) whilst the remainder of 437 (51%) from the high risk plus all mothers classified as low risk (28) did not receive the call (control group). Out of all the mothers who took part in the study, 686 (80%) received the 3 months follow up call, the remainder were unreachable. Thus, our analysis included 308 mothers from the intervention group and 378 mothers from the control group. 17 mothers from the control group and 10 mothers from the intervention group accessed other healthcare services (see Figure 2).

### Descriptive Satistics

#### Description of the Analysis Sample

The characteristics of the study sample are shown in Table 1. Out of the 686 mothers with a 3-month follow up call, 308 (45%) received the 7-14-day call. Over 98% of those who received the call expressed that the call was helpful. Of those who found the call helpful, approximately 9% were happy with the call because baby/mother issues were resolved as a result of the call and 91% were just happy to receive a check up call from the nurse.

**Table 1:** Characteristics of MIP who received and did not receive the 7–14-day call.

**Characteristics**	**7-14 day** **call-No** ***N*** **= 378 (55%)**	**7-14 day** **call-Yes** ***N*** **= 308 (45%)**	**7-14 day** **call-Total** ***N*** **= 686 (100%)**
	***N*** **(%)**	***N*** **(%)**	***N*** **(%)**
**Demographics**			
Age category			
<19	27 (7%)	10 (3%)	37 (5%)
19–24	115 (31%)	93 (30%)	209 (30%)
25–34	170 (45%)	148 (48%)	318 (46%)
35+	65 (17%)	57 (18%)	122 (18%)
Employment			
No	330 (87%)	269 (87%)	599 (87%)
Yes	48 (13%)	39 (13%)	87 (13%)
Education			
completed grade 12	164 (43%)	128 (42%)	292 (43%)
has not completed grade 12	214 (57%)	179 (58%)	393 (57%)
unknown		1(0%)	1(0%)
**Health related factors**			
HIV status			
Negative	291 (77%)	235 (76%)	526 (77%)
Positive	87 (23%)	73 (24%)	160 (23%)
Hypertension			
No	353 (93%)	282 (92%)	635 (93%)
Yes	25 (7%)	26 (8%)	51 (7%)
Mom health condition			
No	373 (99%)	306 (99%)	679 (99%)
Yes	5 (1%)	2 (1%)	7 (1%)
Mental health risk			
No	295 (78%)	223 (72%)	518 (76%)
Yes	83 (22%)	85 (28%)	168 (24%)
**Pregnancy factors**			
Four of more pregnancies			
No	306 (81%)	239 (78%)	545 (80%)
Yes	71 (19%)	68 (22%)	139 (20%)
unknown	1 (0%)	1 (0%)	2 (0%)
PV bleeding during pregnancy			
No	376 (99%)	305 (99%)	681 (99%)
Yes	2 (1%)	2 (1%)	4 (1%)
unknown		1 (0%)	1 (0%)
Antenatal booking			
No	9 (2%)	10 (3%)	19 (3%)
Yes	369 (98%)	298 (97%)	667 (97%)
**Delivery factors**			
Delivered out of facility'			
No	371 (98%)	303 (98%)	674 (98%)
Yes	7 (2%)	5 (2%)	12 (2%)
Complications during labor			
No	358 (95%)	288 (94%)	646 (94%)
Yes	20 (5%)	20 (6%)	40 (6%)
Preterm labor			
No	371 (98%)	299 (97%)	670 (98%)
Yes	7 (2%)	9 (3%)	16 (2%)
Cesarean			
No	274 (72%)	234 (76%)	508 (74%)
Yes	104 (28%)	74 (24%)	178 (26%)
Complications during puerperium			
No	257 (68%)	199 (65%)	456 (66%)
Yes	121 (32%)	109 (35%)	230 (34%)
**Baby factors**			
Low birth weight (<2.5 kg)			
No	318 (84%)	253 (82%)	571 (87%)
Yes	44 (12%)	38 (12%)	82 (13%)
no live infant	16 (4%)	17 (6%)	33 (5%)
Macrosomic (>4 kg)			
No	366 (97%)	300 (97%)	666 (97%)
Yes	12 (3%)	8 (3%)	20 (3%)
Infant resuscitation			
No	357 (95%)	285 (93%)	642 (98%)
Yes	5 (1%)	6 (2%)	11 (2%)
no live infant	16 (4%)	17 (6%)	33 (5%)
**Other factors**			
Accessed other health services			
No	361 (96%)	298 (97%)	659 (96%)
Yes	17 (4%)	10 (3%)	27 (4%)

As shown in Table 1, 48% of the participants who received the call were between the ages of 25–34 years, and the majority were not formally employed (87%) and had not completed grade 12 (58%). Amongst the health-related factors we found that 73 (24%) were HIV positive with 70 (96%) on ART at the time of delivery, 26 (8%) reported having chronic hypertension, 2 (1%) reported having another health condition and 85 (28%) had one or more affirmative responses to the mental health risk assessment.

For the pregnancy factors 22% had more than four pregnancies, 1% experienced PV bleeding during pregnancy and 3% did not have an antenatal booking. Amongst the delivery factors we find five (2%) delivered out of facility, 20 (6%) reported experiencing a problem during labor, 74 (24%) had a cesarean delivery, 109 (35%) experienced puerperium problems. For the baby factors 38 (13%) babies were low birth weight, 8 (3%) babies were macrosomic, and six (2%) babies needed resuscitation at delivery. Amongst those who received the 7-14-day call, 10 (3%) accessed other health services. Our study recorded 31 stillbirths, 14 early neonatal deaths and four post discharge deaths.

#### Factors Associated With Access to Other Health Services

Table 2 shows results for the bivariate and multivariable logistic regression analysis of factors associated with access to other health services. In the unadjusted model having hypertension (Odds Ratio [OR] 3.03; Confidence Interval [CI] 1.10–8.37), mental health risk (3.02; 1.39–6.56), PV bleeding during pregnancy (26.24; 3.55–193.94), problem during labor (3.01; 0.99–9.17) and having a cesarean delivery (0.35; 0.10–1.16) were significantly (*p*-value < 0.05) associated with access to other health services. Although not statistically significant, the odds of accessing other health services were lower amongst those who received the 7-14-day call from the research nurse (0.83; 0.38–1.82).

**Table 2:** Logistic regression of factors associated with access to other health services.

**Characteristics**	**Unadjusted**			**Adjusted**		
	**OR**	***p*** **-value**	**95% Conf Int**	**OR**	***p*** **-value**	**95% Conf Int**
**7-14-day call**						
**No**	**1**					
**Yes**	**0.83**	**0.647**	**0.38–1.82**			
Age	1.01	0.77	0.95–1.07			
Employment						
No	1					
Yes	1.21	0.734	0.41–3.58			
Education						
completed grade 12	1					
has not completed grade 12	1.08	0.840	0.50–2.37			
HIV status						
Negative	1					
Positive	1.40	0.431	0.60–3.27			
Hypertension						
No	1			1		
Yes	3.03	0.033	1.10–8.37	3.28	0.039	1.06–10.10
Mother mental health						
No	1			1		
Yes	3.02	0.005	1.39–6.56	2.82	0.013	1.24–6.38
Four of more pregnancies						
No	1			1		
Yes	0.48	0.235	0.14–1.61	0.35	0.097	0.10–1.21
PV bleeding during pregnancy						
No	1			1		
Yes	26.24	0.001	3.55–19.94	18.33	0.014	1.79–187.61
Delivered out of facility						
No	1					
Yes	2.27	0.442	0.28–18.21			
Complications during labor						
No	1			1		
Yes	3.01	0.052	0.99–9.17	4.40	0.019	1.28–15.13
Preterm labor						
No	1					
Yes	1.65	0.634	0.21–12.98			
Cesarean						
No	1			1		
Yes	0.35	0.086	0.10–1.16	0.22	0.023	0.06–0.81
Complications during puerperium						
No	1					
Yes	1.38	0.420	0.63–3.03			
Macrosomic (>4 kg)						
No	1					
Yes	1.30	0.800	0.167–10.05			
Resuscitation						
No	1					
Yes	2.47	0.400	0.304–20.04			

*The primary exposure of interest (7–14-day call) is highlighted in bold*.

In the adjusted model we find that hypertension (3.28; 1.06–10.10), mental health risk (2.82; 1.25–6.38), PV bleeding during pregnancy (18.33; 1.79–187.61), problem in labor (4.40; 1.28–15.13) were positively, significantly associated (*p*-value < 0.05) with access to other health services. Four or more pregnancies (0.35; 0.10–1.21) and having a cesarean delivery (0.22; 0.06–0.81) were negatively, significantly associated with access to other health services.

#### 7-14-Day Call and Post-Discharge Deaths

Our study recorded 14 early neonatal deaths and four post discharge deaths (further referred to as baby A-D). All mothers for the post discharge deaths were discharged in <24 h and none of the mothers were referred for additional care or follow up at discharge. Baby A was born a twin and died a day after discharge. No problems were identified in either baby. Baby B was born with a low birthweight and died after the 3-6-day visit. During the 7-14-day call the mother reported that the baby experienced convulsions and breathing problems after their return from the 3-6-day checkup. The mother reported that baby died soon afterward, without receiving healthcare. Baby C died between 7 and 14 days after discharge. The hospital file review showed that the baby was referred to the hospital twice for respiratory distress and grunting noise. The first referral was during the 3-6-day visit and the second referral was during an additional non-routine visit that took place 4 days after the 3-6-day visit. During the 7-14-day call the mother reported that baby C was transferred to the hospital were the condition worsened and the baby died. Baby D was born with a low birthweight and died 2-6-weeks after discharge. The mother reported having attended the 3-6-day visit at their health facility and being satisfied with this visit. No problems were identified during the 7–14-day call. During the 3 months follow-up the mother reported that the baby had experienced feeding problems. Three of the post discharge deaths were before the 7-14-day call and the 7-14-day call was not able to identify high risk in the last baby death.

#### 3-to-6-Day Visit and Baby Post-Discharge Death

During the 7-14-day call the research nurse administered a checklist that included a review of the care that the MIPS received at their 3-6 days facility visits. Out of the 417 mothers who received the call 390 reported attending their 3-6-day visit. Overall, the baby checks were well completed, although 10% were not weighed. The mother only reported being asked about her mood in 5% of cases, and 18% of those with a wound report no wound check. Three of the four post-discharge deaths were after the 3-6-day visit. All three mothers attended their 3-6-day visit, two out of the three mothers reported being satisfied with the visit and all baby checks were performed (see Table 3). All three mothers reported that the mother's mood was not asked about or discussed. One of the three babies was referred to the hospital during the call for respiratory distress and grunting noise.

**Table 3:** Description of 3-to-6-day visit and baby post-discharge death.

**Variable**	**Baby post-discharge death**				**Overall**	
	**No (*****N*** **= 414)**		**Yes (*****N*** **= 3)**			
	**No, *N* (%)**	**Yes, *N* (%)**	**No, *N* (%)**	**Yes, *N* (%)**	**No, *N* (%)**	**Yes, *N* (%)**
Did the mother attend the 3-6-day visit at their health facility?	26 (6%)	387 (94%)	0 (0%)	3 (100%)	26 (6%)	390 (94%)
Was the mother satisfied with the 3-6-day facility visit?	2 (1%)	385 (99%)	1 (33%)	2 (67%)	3 (1%)	387 (99%)
**Mother checks**						
Were vital signs done at the visit? (blood pressure)	45 (12%)	342 (88%)	0 (0%)	3 (100%)	45 (12%)	345 (88%)
Was the mother examined at the visit?	33 (9%)	353 (91%)	0 (0%)	3 (100%)	33 (9%)	356 (91%)
Did they ask about vaginal bleeding and discharge at the visit?	45 (12%)	342 (88%)	0 (0%)	3 (100%)	45 (12%)	345 (88%)
Was the Cesarean / perineal wound checked? *	46 (18%)	214 (82%)	0 (0%)	2 (100%)	46 (18%)	216 (82%)
Were the breasts and breastfeeding asked about?	6 (2%)	381 (98%)	0 (0%)	3 (100%)	6 (2%)	384 (98%)
Did they ask the mother about her mood?	367 (95%)	20 (5%)	3 (100%)	0 (0%)	370 (95%)	20 (5%)
**Baby checks**						
Was infant care and feeding discussed?	21 (6%)	359 (94%)	0 (0%)	3 (100%)	21 (5%)	362 (95%)
Was the baby examined?	11 (3%)	368 (97%)	0 (0%)	3 (100%)	11 (3%)	371 (97%)
Was the baby weighed?	37 (10%)	343 (90%)	0 (0%)	3 (100%)	37 (10%)	346 (90%)
**Clinic referral**						
Was the mother referred by the clinic for any services other than the routine 6-week visit	382 (99%)	5 (1%)	3 (100%)	0 (0%)	385 (99%)	5 (1%)
Was the baby referred by the clinic for any services other than the routine 6-week visit	370 (97%)	10 (3%)	2 (67%)	1 (33%)	373 (97%)	11 (3%)

## Discussion

Our study found that that the 7-14-day call did not have a statistically significant impact on additional service utilization. There was, however, non-significant lower odds of accessing additional services, which may point to needs being met during the 7-14 days call. Important factors we found to impact additional service utilization were hypertension, mental health risk, PV bleeding during pregnancy and complications during labor. We also found that the call was not able to identify risk in the one post-discharge infant death that occurred after 7-14-days. Whilst three of the four post discharge deaths were before the 7-14 day-call, three of the deaths were after the 3-6-day visit. It would also appear the 3-6-day visit was able to identify risk in one of the three post-discharge deaths. Our study was able to capture the content of these visits, however, we were unable to assess the quality.

Various studies have shown the effectiveness of postnatal care interventions on mother and baby outcomes (21, 22). It provides health practitioners an opportunity to detect and treat mother and baby health conditions whilst also allowing the mother an opportunity to receive guidance on mother and baby care practices. Although the 7-14-day call intervention did not have a statistically significant impact on additional service utilization, in our study most mothers (98%) expressed appreciation for the call and found the call to be helpful, likely because of the reassurance provided. In contexts with minimal support, even minor interventions can be therapeutic for the mother. Our study finds that of those who found the call helpful, ~91% were happy to receive a check up call from the nurse. The call was also useful in that it was able to identify mother and baby problems and make referrals where necessary. ~9% of those who found the call helpful reported being happy because the call helped resolve mother/baby issues.

The study also finds various other factors that are associated with the use of additional postnatal care. Various studies have assessed the determinants of postnatal care service utilization (23) but studies that seek to establish the determinants of additional postnatal care use are scarce. Whilst the factors that influence postnatal care service utilization varies between settings, previous studies find that factors such as education, age, socioeconomic status, place of delivery and awareness about postnatal care are associated with postnatal care service utilization (23).

Health related factors found to be statistically associated with access to other health services in our study were hypertension and mental health risk. Previous research has shown that hypertension is one of the top three leading causes of maternal mortality in sub-Saharan Africa (24). Therefore, active follow up and improved access to health care by hypertensive mothers would have a positive impact on their health. Our study further showed that mothers with a mental health risk were more likely to access other additional health care services. Consistent with previous studies, PV bleeding during pregnancy was also associated with access to health services (25). Mothers that experienced labor problems such as preterm labor and induction were more likely to access additional health care services. This finding is supported by studies elsewhere which find that women who experience delivery complications are more likely to access postnatal care (26, 27). Our study finds that women who had four or more pregnancies were less likely to access other healthcare services. A study in Nepal finds that women with three or more children are less likely to have had postnatal care (28). This could be related to the perceived postnatal experience obtained from previous child births. Contrary to other studies, we find birth by cesarean section was negatively associated with access to other health care services (26, 27). An explanation for this could be that over 96% of those who reported delivery by cesarean section also reported attending their 3-6-day visit, during which the cesarean wound was checked. Therefore, mothers received the care they needed as part of routine postnatal care services.

Our study recorded 14 early neonatal deaths and four post discharge deaths (20 per 1000). This is below the inpatient neonatal mortality rate in Ba-Phalaborwa sub-district which was estimated to be 23 per 1,000 live births in 2016/17 (18).

As previous literature indicates, some mothers do not attend their recommended routine postnatal visits. The District Health Barometer indicates that in 2016/2017 approximately 82% of women in Ba-Phalaborwa sub-district had their postnatal visit within 6 days, (18). In our study we find that approximately 94% of mothers attended their 3–6-day visit. This was high in comparison to data from the sub-district.

Care of the newborn at the 3-6-day visit should include weighing, examination of the cord and a feeding check to ensure the baby is well. In our study we find that during the 3-6-day postnatal visit most postnatal checks were adhered to except mental health. Although studies have shown that postnatal depression is linked with poor child growth (29, 30) the majority of mothers in our study were not asked about their mood during these visits. Discussions around mental health and appropriate screening should be emphasized as a means to protect and improve mother and baby health.

Despite the importance and novelty of our study it is subject to some limitations. Our study is not nationally representative as data used was collected in rural district in Mopani. Our participants are therefore not from diverse backgrounds and mostly of low socioeconomic status. Despite this limitation our study does provide useful insights for interventions targeting at improving postnatal care and health outcomes. It is also important to note that although the study took place during the COVID-19 pandemic, antenatal care and data collection was not impacted by the restrictions in place at the time (31).

## Conclusion

The postnatal period has often been described as a critical opportunity to save mothers and newborns through the provision of effective health care. The study provides insights into some of the outcomes within the postnatal period and the factors associated with accessing additional health services. Although we find that the 7-14-day call did not have a statistically significant impact on additional service utilization, our study shows that hypertension, mental health risk, labor problems and delivery problems were associated with additional postnatal health care access and mothers who received the call do express gratitude at having received this check-up. There is likely a need to mandate additional postnatal care services for women and infants with certain pregnancy and labor-associated issues, however further work is needed to refine these conditions and the risk factors that cause them. This would allow for these women and their infants to be screened prior to discharge and classified into those requiring additional care and those who can have the traditional standard of care. Additional postnatal care can and should be provided in facility as well as in the home to ensure mothers receive sufficient support in the first 6 weeks following delivery.

## Data Availability Statement

The raw data supporting the conclusions of this article will be made available by the authors, without undue reservation.

## Ethics Statement

The studies involving human participants were reviewed and approved by Ethical approval for data collection was obtained from the Human Sciences Research Council Research Ethics Committee REC 5/21/08/19 and the Limpopo Provincial Health Research Committee (ref: LP-201909-012). Written informed consent to participate in this study was provided by the participants' legal guardian/next of kin.

## Author Contributions

CM: data analysis and manuscript drafting. JD: review of the data analysis and critical review of the manuscript. HS and JM: critical review of analysis and manuscript. KR: review of study design, data analysis and critical review of the manuscript. All authors provided approval for the version to be published.

## Funding

This study has been funded by the South African Medical Research Council, linked to the Mphatlalatsane Project.

## Conflict of Interest

The authors declare that the research was conducted in the absence of any commercial or financial relationships that could be construed as a potential conflict of interest.

## Publisher's Note

All claims expressed in this article are solely those of the authors and do not necessarily represent those of their affiliated organizations, or those of the publisher, the editors and the reviewers. Any product that may be evaluated in this article, or claim that may be made by its manufacturer, is not guaranteed or endorsed by the publisher.

## Abbreviations

MLMH: Maphutha L Malatjie Hospital
WHO: World Health Organization
MMR: Maternal mortality rate
NMR: Neonatal morality rate
SDG: Sustainable Development Goals
CHWs: Community health workers
MIPs: Mother infant pairs.
